# Socio-Demographic Characteristics of Health Care Workers and Hepatitis B Virus (HBV) Infection in Public Teaching Hospitals in Khartoum State, Sudan

**DOI:** 10.5539/gjhs.v4n4p37

**Published:** 2012-05-21

**Authors:** Taha Ahmed Elmukashfi, Omer Ali Ibrahim, Isam Mohamed Elkhidir, Abdelgadir Ali Bashir, Mohammed Ali Awad Elkarim

**Affiliations:** 1 Department of Community Medicine, Faculty of Medicine, University of Khartoum, Khartoum State, Sudan; 2 Department of Econometrics and Social Statistics, Faculty of Economics, University of Khartoum, Khartoum State, Sudan; 3 Department of Medical Microbiology and Parasitology, Faculty of Medicine, University of Khartoum, Khartoum State, Sudan; 4 Khartoum State Ministry of Health, Khartoum State, Sudan; 5 Department of Community Medicine, Faculty of Medicine, University of Khartoum, Khartoum State, Sudan

**Keywords:** HBV markers, health care workers, public teaching hospitals, socio-demographic characteristics, Khartoum State, Sudan

## Abstract

**Background:**

HBV is second to tobacco as a known human carcinogen and the 10^th^ leading cause of death worldwide.

**Objectives:**

To examine the socio-demographic characteristics of health care workers and hepatitis B virus in Public Teaching Hospitals in Khartoum State, Sudan, in 2004.

**Methods:**

It was an observational, cross sectional, facility-based study. A total of 843 subjects were selected. It was conducted through multistage cluster sampling. The clustering was based on: type of hospital (Federal or State) and degree of exposure (type of department). For the analysis, Z-test for single proportion and some non-parametric tests such as Chi-Square test were used.

**Results:**

Among the 843 subjects tested for HBV markers (Anti-HBc, HBsAg, HBsAb, and HBeAg), the prevalence of Anti-HBc, HBsAg, HBsAb, and HBeAg was found to be 57%, 6%, 37% and 9% respectively. Seroprevalence of all HBV markers was found to be statistically significant with demographic factors (P<0.05).

**Conclusion:**

Infection rate, carrier rate and a profile of high infectivity rate were found to be high. The immunity rate was low. There is a significant association between HBV markers and socio-demographic characteristics. Highest rate of infection was found in State Hospitals, South and West regions, married HCWs and HCWs of age group 30-49.

## 1. Introduction

Hepatitis B virus is a widespread disease of public health importance. Nearly 5% of the world populations are currently infected with HBV (Rentería et al., 2002). Infection by HBV is the cause of up to 80% of cases of primary liver cancer, the single most important cause of cancer mortality in the world. In countries where HBV carrier rates either reach or exceed 10%, hepatitis BV may account for up to 3% of total mortality ascribed to HBV infection. One third of HBV chronic carriers who survive for at least 30 years after diagnosis are expected to die due to chronic sequelae of the infection, i.e., cirrhosis and/or primary liver cancer (Rentería et al., 2002), (Lavanchy, 2004). Serologic markers for HBV were detected in 68% of sexually active heterosexuals in Port Sudan and Suakin (McCarthy et al., 1989). In Khartoum, a case - control study was done showed HBsAg positive in 67% of patients with hepatocellular carcinoma and in 4% of control (Itoshima et al., 1989). In Gezira State, Sudan (Khalwat & Salem), HBsAg was 18.7% and seropositivity for any HB markers (HBsAg, Anti-HBs, or anti-HBc) was 63.9% (Hyams et al., 1989). In a cross sectional study carried in Juba, South Sudan, prevalence of HBsAg was 26% and that of Anti-HBcore was 67% (McCarthy et al., 1994).

## 2. Study Area

Khartoum State is one of the 26 states of the Sudan. Khartoum is the political capital and commercial center of the Sudan, with an area of 20140 Km^2^, it lies between latitudes 15.10 to 16.30 degrees north and longitudes 31.35to 34.20 degrees east. The most prominent feature is the Blue and White Niles confluence at the center of the city where the main Nile River is formed meandering up to the Mediterranean Sea. The state is divided into seven localities. Sex ratio is found to be 410 males per 1000 population (Male: female ratio is 1:1). Average size of the households is 6.1 persons per house. Children under five account for 15.1% (higher in the rural areas 14.3%), while those under 14 years of age constitute 37.6%. The working age (15-59) accounts for 57.9% of the total population (Sudan National Population Council, 1993 census).

## 3. Methods

This study was an observational, cross sectional, facility-based study. It was conducted at Public Teaching Hospitals in Khartoum State in the year 2004. A total of 843 subjects were selected, using multistage cluster sampling. The clustering was based on type of hospital (Federal or State) and degree of exposure (type of specialty). To increase the internal homogeneity of the groups, the hospitals were further divided according to whether they have all specialties, two specialties, or one specialty, to reflect the degree of exposure. Informed consent from the selected health care workers (HCWs) obtained. A pre-tested, pre-coded questionnaire was used to collect socio-demographic. 5 ml of venous blood were collected using 10 ml vacutainer. Sera were separated and stored at - 20° centigrade, until testing. ELISA was used to screen for anti-HB core total. Reactive specimens for anti HB core were tested for HBs Ag. Reactive specimens for HBs Ag were tested for HBe Ag. Vaccinated HCWs and part of the reactive specimens for anti- HB core but non-reactive for HBs Ag were tested for Anti-HBs.

Data was processed using the statistical package for social sciences (SPSS), version 15. For the analysis, Z-test for single proportion and some non-parametric tests such as Chi-Square test were used, P-value of <0.05 was considered statistically significant.

## 4. Results

A total of 843 HCWs were interviewed and blood sample was collected; 628 (74.5%) from Federal Teaching Hospitals, and 215 (25.5%) from State Teaching Hospitals. The age group 30-49 years represents 58.4% of the subjects, followed by the age group less than 30 (30.7%), and only (10.9%) for more than 50. Regarding gender representation, 366 (43.4%) were males and 477 (56.6%) were females. Four hundred and sixty (54.6%) HCWs were married. Concerning education, 269 (31.9 %) HCWs were university graduates, 214 (25.4 %) high secondary education and five (0.6%) Quranic education (khalwa). The health care workers were originally from the Central 288 (34.2%), Western 235 (27.9%), Northern 212 (25.1%), Southern 86 (10.2%) and Eastern Region 22 (2.6%) of the Sudan.

The findings of the Z-test for single proportion were as follows:

### 4.1 Infection Rate (+ve Anti-HBc)

Four hundred and seventy seven (57%) of the respondents had evidence of past or ongoing HBV infection. (95% CI: 54% - 60%).

### 4.2 Carrier Rate (+ve HBsAg)

Twenty seven (6%) of the tested respondents represented the HBV carriers with a low infectivity profile. (95% CI: 5.9% - 6.1%)

### 4.3 Immunity Rate (+ve Anti-HBs)

Forty six (37%) of the tested individuals were positive for Anti-HBcore. (95% CI: 36.2% - 37.7%)

### 4.4 A Profile of High Infectivity Rate (+ve HBeAg)

Out of 27 carriers, three (9%) were positive (95% CI: 6.9% - 11.1%).

### 4.5 Socio-demographic Characteristics Associated with High Prevalence of Infection (positive Anti-HBcore)

Table 1 indicates that the highest infection rate occurred within State Teaching Hospitals with a rate of 62.8% compared with 54.5% for Federal Teaching Hospitals. With regard to the original place of residence, the Southern and the Western States obtained the highest prevalence, 67.4% and 65.5% respectively. The least prevalence (48.6%) was found in the Northern State. Among the married HCWs 59.8% were positive and for unmarried they were 53.0%. For educational, the highest prevalence of Anti-HBc was associated with Quranic school (Khalwa) education (60%), followed by intermediate (58.4%), postgraduate (56.8%) and illiterate (37%).

**Table 1:** Demographic characteristics of HCWs and the HBV markers (all values in percentages)

Characteristics	N	Anti-HBcore (N=843)		HBsAg (N = 488)		HBeAg (N = 32)		Anti-HBsAg (N=123)	
		N	%	N	%	N	%	N	%
		+		+		+		+	
**Hospital**		*							
Federal	628	342	54.5	24	6.6	37	8.0	2	37.8
State	215	135	62.8*	4	2.8	1	36.0	9	14.3
**Sex**								
Male	366	214	58.5	16	7.3	2	11.1	26	41.3
Female	477	263	55.1	12	4.4	1	7.1	20	33.3
**Age**				*					
< 30 years	259	131	50.6	7	5.2	2	22.2	19	46.3
30 – 49	492	292	59.3	16	5.4	1	5.9	24	33.8
50 +	92	54	58.7	5	7.3	0	0.0	3	27.3
**Residence**		*							
South	86	58	67.4	6	10.2	0	0.0	1	33.3
North	212	103	48.6	4	3.8	1	20	17	53.1
East	22	12	54.5	1	8.3	0	0.0	3	75.0
West	235	154	65.5	13	7.7	2	13.3	5	17.9
Center	288	150	52.1	4	2.6	0	0	20	35.7
**Marital Status**		*							
Married	460	275	59.8	17	6.0	1	5.3	18	30.0
Unmarried	381	202	5.30	11	53.0	2	15.4	28	44.4
**Education**		*						
Illiterate	97	36	37.2	4	5.5	0	0.0	1	16.7
Khalwa	5	3	60.0	0	0.0	0	0.0	0	0.0
Primary	59	30	55.9	4	11.8	0	0.0	1	25.0
Intermediate	125	73	58.4	6	6.9	0	0.0	5	35.7
Secondary	214	115	53.7	8	6.7	2	20.0	2	10.0
University	269	140	52.0	6	4.2	1	14.3	30	48.4
Postgraduate	74	42	56.8	0	0.0	0	0.0	7	43.8
**Duration of work**								
< 1 year	73	35	47.9	0	0.0	0	0.0	2	40.0
1 year.	101	55	54.5	3	5.5	1	33.3	10	47.6
5 years	232	131	56.5	7	5.2	1	10.0	13	40.6
10 years	137	73	53.3	3	4.1	0	0.0	4	25.0
15 years	107	63	58.9	5	7.8	1	16.7	9	40.9
> 20 years	193	119	61.8	10	7.4	0	0.0	8	29.6

* Result is significant at P value < 0.05 according to Chi-Square test. * For marital status two cases were missing.

### 4.6 Socio-demographic Characteristics Associated with High Carrier Rate (HBsAg)

The prevalence of carrier of HBV increased with increase in age: from 5.2% for the age <30 years to 5.4% for the age 30-49 reaching 7.3% for the age group 50+.

## 5. Discussion

Hepatitis BV is a blood borne virus. Infection frequently results in a chronic asymptomatic carrier state for many years before the development of symptomatic liver disease. HBV infected healthcare workers may therefore be unaware of their condition and their potential to infect patients. Healthcare workers, who perform exposure prone procedures, where injury to the worker may result in exposure of the patient’s open tissues to the blood of the worker, are at increased risk of infection with blood borne viruses.

The highest infection rate was observed among the age group 30-49. This was consistent with that recorded by WHO, and with a study done in Canada (WHO, 2008), with regard to the original residency of the HCW, the South and the West Regions obtained the highest infection rate. The North Region showed the least infection rate. This is because the disease was endemic in the South and West regions and possibly other undetected factors. The obtained results were consistent with the findings recorded in Iran; in U.S.A.; and in Juba, Southern Sudan (WHO, 2008; Alavian et al., 2005; Teo & Lok, 2006; McCarthy et al., 1994). Among the married HCWs, positive Anti-HBc was more prominent than among unmarried HCWs. Highest prevalence of Anti-HBc was associated with Quranic School (Khalwa) type of education, followed by intermediate, postgraduate and the least are the illiterate HCWs. The illiterate HCWs had least contact with patients compared to other HCWs while postgraduates were more aware of safety measures. Significant association was found between age, type of hospital, original place of residence, marital status and education of the HCW and infection rate (+ ve Anti-HBcore).

The highest carrier rate of 10.2% was found in the South, followed by 8.3% in the West and 2.6% in the Central State. Demographic characteristics, age and occupation were found to be significantly associated with carrier rate measured by HBsAg (P< 0.05). The findings were similar to that recorded by WHO; a study done in Canada (Rentería et al., 2002; McCarthy et al., 1994), Port Sudan and Suakin, Khartoum, and Gezera, in Sudan (McCarthy et al., 1989; Itoshima et al., 1989; Hyams et al., 1989; Elshafie, 1992). The prevalence of carrier of HBV increased with increase in age: from 5.2% for the age <30 years to 5.4% for the age 30-49 reaching 7.3% for the age group 50+. The highest carrier rate occurred at the age group of 30-49. The lowest carrier rate occurred in the lowest age group i.e. < 30 years old. Concerning the effect of place of residence on the carrier rate, the Southern and the Western Regions recorded the highest carrier rate, while the Central Region has the lowest carrier rate. This is because the disease is endemic in these areas and therefore needs further studies. However, this result was identical with that of the infection rate of HBV infection.

Out of the 27 currently infected individuals, 3 (9%) were considered as a high profile infected people. The P-value of the Z-test for single proportion = 0.000. The highest rate of High Profile of Infectivity occurred in the age group 30-49. The lowest carrier rate occurred in the age group (< 30).

Those who were vaccinated against HBV infection or infected with HBV and developed immunity accounted for 37% (95% CI: 34%-40%). The P-value of the Z-test for single proportion = 0.007.

Completion of vaccination is recommended during training in schools of medicine, dentistry, nursing, laboratory technology, and other allied health professions, before trainees have their first contact with patient’s blood and body fluids. Establishment of an occupational health unit is highly recommended in each hospital. This unit has to implement and monitor the infection control system in the hospital

## 6. Conclusion

The study showed that the infection rate among HCWs in Public Teaching Hospitals in Khartoum State measured by Anti-HBcore was found to be 57%, while the carrier rate measured by HBsAg was 6% and the high profile infectivity rate measured by HBeAg was 9%. The immunity rate measured by Anti-HBsAg was 37%. Among the different demographic characteristics the type of hospital, original residency, marital status, and education; were statistically significant with Anti-HBcore. Increased age was found to be associated with high carrier rate.
